# Toward Elimination of Electrochemical Corrosion in Dental Implants: A Zirconia-Titanium Composite Prototype

**DOI:** 10.7759/cureus.49907

**Published:** 2023-12-04

**Authors:** Alex Pozhitkov, Eric Lindahl, Daniel C Chan

**Affiliations:** 1 Division of Research Informatics, City of Hope National Medical Center, Duarte, USA; 2 Restorative Dentistry, University of Washington School of Dentistry, Seattle, USA; 3 Physics, University of Washington, Seattle, USA

**Keywords:** atomic force microscopy, peri-implantitis, microbial fuel cell, corrosion, titanium, dental implant, osseointegration

## Abstract

Background

Titanium dental implants (e.g., Nobel Biocare, Switzerland) are routinely used as support for dental restoration. Titanium has been the material of choice due to its corrosion resistance and ability to integrate with bone. Nevertheless, corrosion and titanium dissolution do occur. Compared to control, peri-implantitis tissue biopsies have been shown to contain high concentrations of dissolved titanium as well as metal particles. Dissolved titanium species have been found to be associated with the structure/diversity of the subgingival plaque microbiome and the extent of global methylation. Of note, peri-implantitis and peri-implant mucositis are common biological complications of implant therapy. Microorganisms and local inflammation together with a gradient of oxygen have been proven to form an electrochemical fuel cell, which generates the current that flows through the body of the titanium implant. Effectively, the fuel cell reduces oxygen and oxidizes titanium that turns into a soluble form. We are proposing a new zirconia-titanium composite implant design whereby the electrical current is disrupted while other properties are still conducive to osseointegration.

Methodology

Biocompatible zirconia bolts were treated with hydrofluoric acid (HF) and coated with titanium in a vacuum evaporator. The coating was masked with nail polish, and unmasked areas were etched with HF followed by mask removal with a solvent. Microbial challenges were conducted with a volunteer’s plaque. Regular implant (control) and the prototype were inserted into simulated peri-implant environments implemented as a fiberglass sleeve immersed into a growth medium. After a five-day growth, samples were taken and HNO_3_ digested. Dissolved titanium was evaluated by inductively coupled plasma mass spectrometry.

Results

Proof-of-concept implant prototypes were successfully created. Vacuum deposition results in reproducible stable titanium coating. The thickness of the titanium coating was estimated using atomic force microscopy. A microbial challenge revealed that compared to the commercial titanium implant, the new implant prototype showed decreased amounts of corrosion-leached titanium.

Conclusions

We demonstrate a path forward toward a new design of a dental implant, whereby corrosion-induced electrical currents are interrupted resulting in a decreased amount of dissolved titanium.

## Introduction

Titanium dental implants (e.g., Nobel Biocare, Switzerland) have been successfully used as support for dental restoration since the end of the 1970s. Although earlier versions of implants (made of steel) existed, compared to titanium, they lacked integration with the bone (osseointegration). Hence, titanium has been the material of choice ever since due to its ability to integrate with bone and corrosion resistance [1]. Nevertheless, corrosion of the titanium dental implants does occur. The corrosion processes include disruption of the protective oxide layer leading to titanium dissolution [2]. Corrosion-inducing factors include (i) local acidification due to inflammation of peri-implant tissues [3], and (ii) generation of an acidic environment by bacteria, e.g., the release of lactic acid by *Streptococcus mutans* [4]. In addition, chemical agents, such as acidic fluoride solutions, have been associated with corrosion [5]. The electrical conductivity of the implant itself has been implicated in the process of corrosion [6]. Specifically, in the presence of bacteria, a closed circuit is formed between partially oxygenated (anode) and anoxic zones (cathode).

The associations between the detected corrosion products and implant health have been recently investigated. Compared with healthy implants, increased quantities of dissolved titanium were detected in submucosal plaque around implants affected with peri-implantitis [7]. A recent study showed that peri-implantitis tissue biopsies contain high concentrations of titanium compared to controls from periodontitis tissue. Moreover, titanium metal fragments were identified in the peri-implantitis tissue [8]. An association between the dissolved titanium species and the structure/diversity of the subgingival plaque microbiome was established [9].

Another interesting finding was that dissolved titanium concentration was associated with the extent of global methylation independent of the peri-implantitis status. Peri-implantitis and peri-implant mucositis are common biological complications of implant therapy [10]. A recent systematic review revealed that the prevalence of peri-implant mucositis and peri-implantitis ranges from 19% to 65% [11]. This has been confirmed by one of the largest studies performed in Sweden [12].

One way to minimize peri-implantitis and combat corrosion is by ensuring that corrosion-generated electrical currents are blocked. Here, we propose a new dental implant design that combines osseointegration properties of titanium and at the same time offers very high impedance to the corrosion electrical current. The body of the implant is made of a non-conductive material, e.g., zirconia or porcelain ceramic coated with electrically isolated titanium rings. We report preliminary results of a microbiological challenge of the new design with volunteer-derived microbiota in vitro.

## Materials and methods

Biocompatible zirconia bolts (Ceramco Inc., Z10320HEX1.250, 10-32 x 1-1/4" Zirconia Hex Head Bolt) were treated with 15% hydrofluoric acid (HF) for several minutes, followed by a rinse with deionized water, and heating up to 300°C for more than two hours followed by cooling down. Titanium coating was performed in a vacuum evaporator (JEOL Inc.) with a 0.5 mm titanium wire 110.8 mg (99.99% purity) wound around a 0.75 mm tungsten wire (99.95% purity). Stripes were created by masking titanium zones with nail polish, followed by 15% HF etching for approximately 20 seconds, and nail polish removal with methyl ethyl ketone for two hours.

The media for microbial challenges was prepared as follows: trypticase Soy Broth (30 g/L), yeast extract (5 g/L), pH adjusted to 7.2, and autoclaved. Upon cooling down, the media base was supplemented with vitamin K3 stock (0.2 mL/L) and hemin stock (filter-sterilized) (10 mL/L). The hemin stock was prepared by dissolving 50 mg of hemin (Sigma-Aldrich) in 1 mL of 1 M NaOH followed by diluting with 99 mL of deionized water water. Vitamin K3 stock was prepared by dissolving 250 mg of vitamin K3 in 50 mL of 95% ethanol.

Oral microorganisms were obtained as subgingival/interdental plaque samples derived from the authors who voluntarily agreed to provide samples. Specifically, a pipette tip was used by the volunteers to collect their plaque from around the lower first molar and second pre-molar. The plaque was suspended in the growth medium. Using a syringe, 3 mL of bacterial suspension was added between the fiberglass sleeves and the screws (details of the fiberglass/implant setup are presented in the Results). Subsequently, fresh sterile media was poured into the beaker (between the beaker and the sleeves). Microbial growth was conducted for five to seven days at 37°C in a covered water bath allowing gas exchange and presumably ~100% humidity. Upon completion of the microbial challenge, 1 mL of the medium was harvested near the outside of the fiberglass sleeve.

Similar to our earlier research [6], titanium measurements were conducted by inductively coupled mass spectrometry (ICP-MS) using the Agilent 7500CE mass spectrometer. The organics were digested in a solution containing 50:50 (V/V) concentrated HNO_3_:deionized water, which were Fisher trace-metal grade and Barnstead Nanopure ≥18 MOhm/cm, respectively. The digestion solution also contained trace amounts of HF and 10 ppm Tb as a recovery standard (BDH reagents). Each sample was brought to 5 mL with the digestion solution, followed by open-vessel microwave digestion at the following settings: power 800 W, 100%, ramp 15 minutes to 100°C, and hold for 45 minutes (Mars Xpress, CEM). The digested samples were diluted up to 25 mL with deionized water. 45Sc was used as an internal standard for calibration, whereby the calibration standards (0.01-100 ppb) were at the same final acid concentration as the samples. The standards were prepared from single-element commercial standards (Ultra Scientific; certified reference material) and checked with an alternate calibration standard prepared from a different lot or vendor (BDH). Polyatomic interferences were eliminated by running the instrument in He mode. The detection limit was 0.01 ng/mL. Finally, the results were adjusted for the process blank values.

A transmission electron microscopy (TEM) mesh 3HGC 500 (Ted Pella Inc.) was used to create a regular pattern on mica disks. Titanium wire was wrapped along the top turns of the tungsten basket EVB12A3030W (Ted Pella Inc.). The basket was heated at 60 VAC for less than one minute until the titanium wire disappeared. The distance between the top turn of the basket and the mica disks was 100 mm. The Bruker Multimode 8 atomic force microscope (AFM) was used to measure the height profile of titanium patches on the surface of mica disks.

## Results

Implant prototype

**Figure 1:**
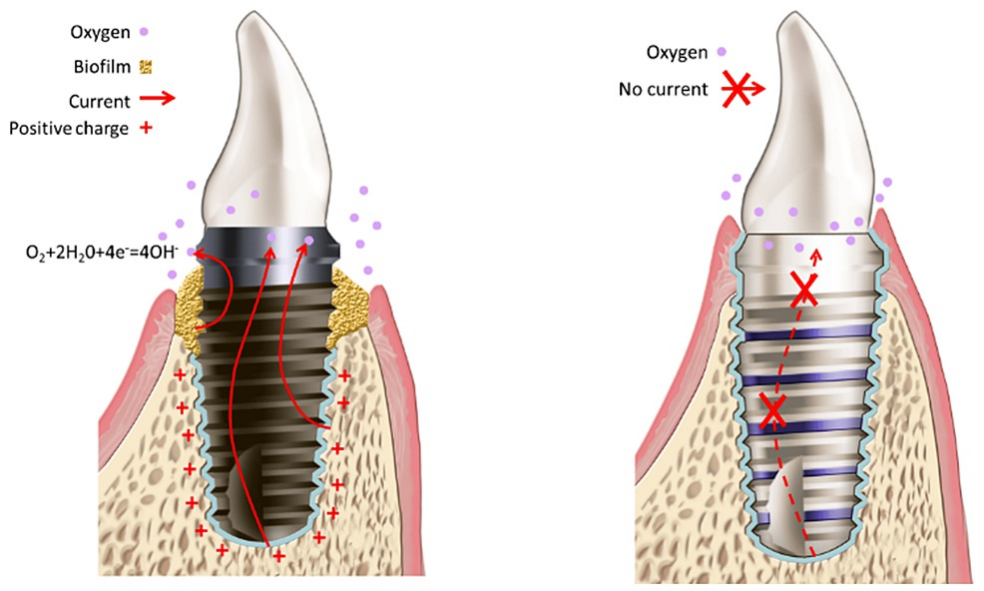
Left: a presumptive electrochemical process involving a titanium dental implant. Electrical charges flow from the lower part toward the top, where oxygen reduction occurs. The lower parts of the implant give off electrons and dissolve as titanium ions. Right: proposed new design, whereby the non-conductive body of the implant is coated with electrically disconnected titanium stripes.

The concept behind the implant prototype is depicted in Figure 1, right panel. The body of the prototype is a #10 bolt made of biocompatible zirconia ceramic. The bolt was initially completely coated with titanium in a vacuum evaporation system.

**Figure 2:**
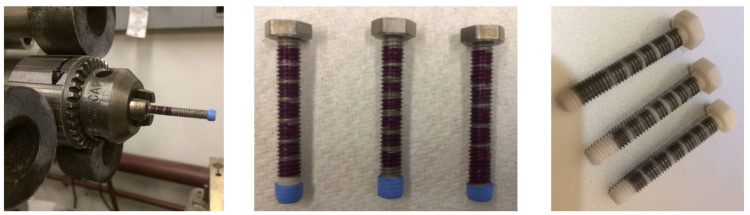
Steps for creating implant prototype. From left to right: (i) placing nail polish mask in a lathe; (ii) masked bolts ready for etching; and (iii) completed prototype.

Electrically insulated stripes were produced by etching a part of the titanium coating with stripes protected by masking. Figure 2 outlines the major steps in the fabrication of the implant prototype.

Microbial challenge

**Figure 3:**
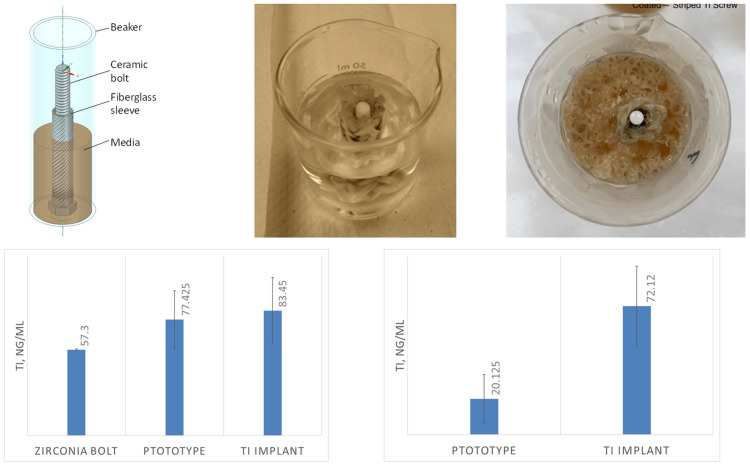
Microbial challenge in liquid media. Top row, from left to right: (i) conceptual diagram of the microbial challenge; (ii) implant prototype placed into the fiberglass sleeve matrix; and (iii) top view of the media after complete microbial growth. Bottom left: concentrations of dissolved titanium detected in the medium. Bottom right: the amount of leached titanium estimated by subtracting the background (see text).

The implant prototype was challenged with a mixed microbial population obtained from a volunteer’s subgingival plaque. The implant prototypes (and controls) were covered with a fiberglass sleeve to simulate the peri-implant space. The rationale behind this approach is that fiberglass would restrict fluid convection, and hence, maintain oxygen gradient, which presumably adequately simulates the peri-implant environment (Figure 3).

Six prototypes, two commercial implants (solid titanium), and one zirconia bolt were subjected to the microbial challenge, which was repeated two times. At the end of the experiment, some of the commercial implants or prototypes appeared tilted or with excess liquid; such specimens were excluded from the analysis (Table 1).

**Table 1:** Titanium concentrations measured after five days of microbial challenge. *: excluded from the analysis.

	Sample ID	Titanium (ng/mL)	Specimen	Notes
Challenge 1	1	57.7	Zirconia bolt	
	2	64.5	Commercial implant	Tilted^*^
	3	132	Commercial implant	Tilted^*^
	4	99.7	Prototype	
	5	82.1	Prototype	Tilted^*^
	6	80.6	Prototype	
	7	88.7	Prototype	
	8	84.6	Prototype	
	9	202	Prototype	Tilted^*^
Challenge 2	10	56.9	Zirconia bolt	
	11	99.2	Commercial implant	
	12	67.7	Commercial implant	
	13	62.4	Prototype	
	14	39.1	Prototype	Excess liquid^*^
	15	70	Prototype	
	16	44.1	Prototype	Excess liquid^*^
	17	73.5	Prototype	
	18	59.9	Prototype	

The concentration of titanium was previously measured in the media at 11.33 ± 2.08 ng/mL (mean ± s.d.). To estimate the amount of leached titanium, the value of the zirconia bolt specimen was subtracted from the value of the prototype specimen. This procedure accounts for the titanium background of the medium and the uncoated zirconia bolt. Moreover, 11.33 ng/mL was subtracted from the value of the commercial implant specimen to account for the titanium background concentration in the medium. The results are graphically shown in Figure 3 bottom left; two-tailed t-test p = 0.002 assuming equal variances (if variances are assumed unequal, p = 0.162).

Thickness of the coating

Evaporating a comparable amount of titanium onto mica disks through a TEM mesh allowed for estimating the thickness of the titanium coating on the prototype. Three different amounts were evaporated onto pairs of disks and the thickness was measured (Table 2).

**Table 2:** Thickness of titanium patches on mica disks as an estimation of the zirconia bolt coating.

Trial	Titanium, mg	Thickness, nm
1	83.9	114
1	83.9	133
2	36.1	139
2	36.1	113
3	161.5	311
3	161.5	309

**Figure 4:**
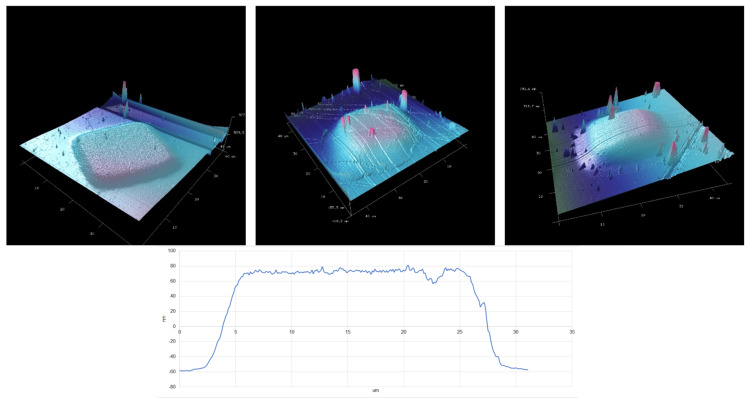
Atomic force microscopy estimation of the thickness of the titanium coating. Top row: trials one to three; bottom row: a cross-section of a titanium patch.

Figure 4 shows the AFM images of the titanium patches from trials one to three. Based on the measurements, we estimate that the thickness of the titanium film of our implant prototypes was about 120 nm because 0.1108 g of titanium used in the coating was close to trial one.

## Discussion

The proposed design for the implant prototype is based on the notion that a microbial fuel cell is formed in the peri-implant space [6] (Figure 1). The electrochemical process within this cell is a reduction of oxygen (cathode) and oxidation of organics and titanium (anode). It is important to understand that this fuel cell does not generate any usable voltage, but rather is “shorted” by the body of the conductive titanium implant. Peri-implant space is presumably filled with local microenvironments stacked along the axis of the implant. These microenvironments generate electrochemical potential capable of driving the electrical current leading to galvanic corrosion. The proposed design features ring-like titanium zones electrically insulated from one another to prevent the electrical current and therefore the galvanic corrosion. Although the galvanic current may flow along the vertical part of the ring, the concentration differences between the microenvironments along the short vertical portion of the ring are expected to be much smaller compared to the difference between one end of the implant and the other. Therefore, the concentration-driven voltage difference (and therefore the current) through the ring is expected to be much smaller compared to the fully conductive metallic implant. It can be argued that to solve the corrosion problem an inert electrically non-conductive material can be used such as biocompatible zirconia. Nevertheless, the osseointegration capability of zirconia is inferior to that of titanium in a dental implant setting (refer to a comparative review of titanium vs. zirconia [13]). The ring-like titanium zones provide anchoring points for osseointegration. At the same time, each titanium zone resides in not more than one microenvironment, hence, the lateral galvanic currents are diminished and so is the galvanic corrosion.

Taken together, the logic of the new design can be summarized as follows. The new design of the intraosseal dental implant comprises a chemically inert, electrically non-conductive, screw-shaped body coated with electrically separated titanium rings. This design utilizes a well-known property of titanium to integrate with the bone, yet the electrical insulation of the titanium zones prevents the flow of galvanic corrosion currents. Although the importance of galvanic corrosion to the development of peri-implant disease may be disputed, existing evidence suggests that the corrosion does occur. It is yet to be determined whether the corrosion is the cause or a consequence of peri-implantitis, however, the elimination of the corrosion will prevent tissue reactions to its products in the peri-implant space.

The microbial challenge of dental implant materials in vitro is usually done with pure cultures or simple microbial compositions of two to three species [14,15]. We rationalized that a realistic implant environment is much more complex. Our previous work on titanium corrosion employed a volunteer’s dental plaque, and it was shown that the microbial diversity in the in vitro setting is comparable to that of the original plaque [6]. The same approach was undertaken in this study, although the volunteer was different. Ideally, various plaques obtained from healthy individuals and those suffering from peri-implantitis should have been tested; however, such analysis was beyond the scope of this preliminary report.

The belief that implants are a panacea to replace missing teeth and yield a better prognosis has been rejected in comparative studies and systematic reviews. Although the reasons for failures are complex and multifaceted, we attempted to focus on the unique electrochemical process in our study. This important concept has not been fully evaluated. Future research in refining the zirconia-titanium composite implant will involve reducing the size of titanium rings while increasing their number.

There are some limitations of our study. First, the duration of the microbial challenge was short. It would be beneficial to increase the duration to up to several weeks. However, the long-term microbial challenge would require some form of continuous growth medium supply and effluent collection (for titanium analysis). Second, the fiberglass proxy for the bone socket may be considered quite a remote simulation. In future research, a cadaver or an animal bone could be used to not only evaluate the extent of corrosion but also assess the strength of zirconia-titanium binding.

## Conclusions

We demonstrated a path forward toward a new design of a dental implant, which combines the best of the two worlds: the inertness of zirconia ceramic and the osseointegrative capability of titanium. Practical steps in the fabrication of such an implant were presented in our report. When challenged with complex oral microbiota, a decreased leaching of titanium from the prototype was observed compared to a commercial titanium implant. We believe that the interrupted corrosion-induced electrical currents will be key to the in vivo success of the new implant design.
